# *Chryseobacterium indologenes *infection in a newborn: a case report

**DOI:** 10.1186/1752-1947-5-10

**Published:** 2011-01-14

**Authors:** Gema Calderón, Esther García, Pilar Rojas, Elisa García, Marisa Rosso, Antonio Losada

**Affiliations:** 1 Neonatology Unit, 'Virgen del Rocío' University Children's Hospital, Seville, Spain

## Abstract

**Introduction:**

*Chryseobacterium indologenes *is an uncommon human pathogen. Most infections have been detected in hospitalized patients with severe underlying diseases who had indwelling devices implanted. Infection caused by *C. indologenes *in a newborn has not been previously reported.

**Case presentation:**

We present a case of ventilator-associated pneumonia caused by *C. indologenes *in a full-term Caucasian newborn baby boy with congenital heart disease who was successfully treated with piperacillin-tazobactam.

**Conclusion:**

*C. indologenes *should be considered as a potential pathogen in newborns in the presence of invasive equipment or treatment with long-term broad-spectrum antibiotics. Appropriate choice of effective antimicrobial agents for treatment is difficult because of the unpredictability and breadth of antimicrobial resistance of these organisms, which often involves resistance to many of the antibiotics chosen empirically for serious Gram-negative infections.

## Introduction

*Chryseobacterium *spp. are Gram-negative bacilli widely distributed in soil and water. In hospital environments, they have been recovered from water systems and humid surfaces. Infections caused by *Chryseobacterium indologenes *are rare, but have been reported as a cause of serious infections in adult immunosuppressed patients. To the best of our knowledge, infection caused by *C. indologenes *in a newborn has not been previously reported.

## Case presentation

Our patient, a full-term Caucasian newborn baby boy with congenital heart disease (double-outlet right ventricle, mitral atresia and hypoplastic aortic arch) remained intubated and under mechanical ventilation from the seventh day of life due to hemodynamic deterioration. Then, 20 days later, he deteriorated clinically with worsening fever, intense leukocytosis, increase of acute-phase reactants and pulmonary infiltrate on chest radiograph. Empiric antibiotic therapy with meropenem and vancomycin was given. Bacteriological blood, cerebrospinal fluid and urine culture test results were negative. *C. indologenes *was isolated from a tracheobronchial secretion sample obtained by endotracheal aspiration. Treatment was discontinued at 10 days on clinical improvement. Then, five days later, he again developed fever and pulmonary infiltrate on chest radiograph. *C. indologenes *was again isolated from respiratory samples obtained by bronchoalveolar lavage (BAL). No other microorganisms were isolated from the BAL sample. The bacteria were susceptible *in vitro *to fluoroquinolones, cefepime, piperacillin-tazobactam and co-trimoxazole with intermediate susceptibility to third-generation cephalosporins; it was resistant to meropenem, imipenem, aztreonam, sulbactam-ampicillin and aminoglycosides. Antibiotic therapy with piperacillin-tazobactam was given and continued for 14 days. Our patient continued to do well up to the time of surgery for the repair of the congenital heart disease two months later.

## Discussion

The genus *Chryseobacterium *belongs to the family Flavobacteriaceae. Six species of *Chryseobacterium *are more commonly isolated from clinical specimens: *C. meningosepticum*, *C. odoratum*, *C. multivorum*, *C. breve *and group IIb *Chryseobacterium *spp., which includes *C. indologenes *and *C. gleum. Chryseobacterium *spp. are Gram-negative, aerobic, non-fermentative, oxidase-positive and catalase-positive non-motile bacilli that produce a distinct yellow to orange pigment [1]. They are widely distributed in nature and found primarily in soil and water. They are not normally present in the human microflora [1,2]. They can survive in chlorinated waters, and in the hospital environment they exist in water systems and wet surfaces and serve as potential reservoirs of infection. Colonization of patients via contaminated medical devices such as respirators, endotracheal and tracheostomy tubes, humidifiers, incubators for newborns and syringes has been documented previously [2,3]. Contaminated surgically implanted devices such as intravascular catheters and prosthetic valves have also been reported [4]. *Chryseobacterium *infections in humans are usually acquired nosocomially and are frequently associated with the presence of invasive equipment (intra-vascular catheters, endotracheal tubes, prosthetic device) in immunocompromised patients or patients who have received long-term broad-spectrum antibiotics [4,5]. *C. meningosepticum *is the most pathogenic member of the genus; it is an agent of neonatal meningitis with mortality rates of up to 57% and is involved to a lesser extent in cases of pneumonia and bacterial sepsis in neonates and adults [6]*C. indologenes *is an uncommon human pathogen. The clinical significance of *C. indologenes *has not been fully established yet because this bacterium has not been frequently recovered from clinical specimens. Reported infections include bacteriemia, ventilator-associated pneumonia, indwelling device-associated infection, pyonephritis, biliary tract infection, peritonitis, lumboperitoneal shunt infection, ocular infections, and surgical and burn wound infections, and infection has been associated with a high mortality rate [4,5,7-13].

In the literature we have found six cases published of infections for C. indologenes in children; all of the patients were older than three months of age [9-13]. Hsueh et al. [9,10] reported three pediatric cases of C. indologenes bacteremia. The first two patients were a one-year-old girl and a five-year-old girl, both receiving chemotherapy for a neoplastic disease and both with indwelling central venous catheters. The third patient was a one-year-old boy with a burn injury who was under mechanical ventilation. The one-year-old boy with burns developed an adult respiratory syndrome and died despite antimicrobial treatment; the other two patients recovered after three days of treatment. Cascio et al. [11] reported on a two-year-old boy with type 1 diabetes mellitus who developed bacteremia. The only medical device present was a peripheral catheter. The patient received antimicrobial treatment with ceftriaxone and recovered after two days.

In 2007, Bayraktar et al. [12] reported on a bloodstream infection in a five-month-old baby. Molecular typing with arbitrarily primed polymerase chain reaction demonstrated the cross-contamination of commercial distillate water. The baby was infected by this water as a result of medical assistance received during hospitalization.

Al-Tatari *et al*. [13] reported on a lumboperitoneal shunt infection in a 13-year-old boy with congenital hydrocephalus successfully treated with trimethoprim-sulfamethoxazole and rifampim.

To the best of our knowledge, our patient's case is the first reported example of infection caused by *C. indologenes *in a newborn. Appropriate choice of effective antimicrobial agents for treatment of infection by *C. indologenes *is difficult because of the unpredictability and breadth of antimicrobial resistance of these organisms, which often involves resistance to many of the antibiotics chosen empirically for serious Gram-negative infections.

*C. indologenes *is often resistant to extended-spectrum penicillins, first-generation and second-generation cephalosporins, ceftriaxone, aztreonam, ticarcillin-clavulanate, chloramphenicol, erythromycin, aminoglycosides, imipenem and meropenem for production of a class B carbapenem-hydrolyzing enzyme.

*C. indologenes *is usually susceptible to piperacillin alone or combined with tazobactam, ceftazidime, cefepime, fluoroquinolones, rifampin and cotrimoxazole, but the *in vitro *susceptibility to these antibiotics should be systematically tested.

Antimicrobial susceptibility data on *Chryseobacterium *spp. remain very limited because this pathogen has rarely been isolated from clinical specimens. The results of the evaluation of a worldwide collection indicate that the newer quinolones (garenoxacin, gatifloxacin, and levofloxacin) may represent the most appropriate antimicrobial agents to treat infections caused by this pathogen. Garenoxacin was the most active quinolone (minimum inhibitory concentration required to inhibit the growth of 50% of organisms (MIC50): 0.12 μg/mL); gatifloxacin (MIC50: 0.25 μg/mL) and levofloxacin (MIC50: 0.5 μg/mL) also inhibited 98.0% of the isolates, and the rate of susceptibility to ciprofloxacin (MIC50: 0.5 μg/mL) was significantly lower. Trimethoprim-sulfamethoxazole showed reasonable activity. Among the β-lactams, the most active agents overall were piperacillin-tazobactam (MIC50: 4 μg/mL; 80.0% susceptibility), piperacillin (MIC50: 8 μg/mL; 74.0% susceptibility), and cefepime (MIC50: 8 μg/mL; 62.0% susceptibility). The carbapenems (6% to 12% susceptible) and the aminoglycosides (8% to 14% susceptible) exhibited poor activity against these pathogens [14].

## Conclusion

*C. indologenes *should be considered as a potential pathogen in newborns in the presence of invasive equipment or on treatment with long-term broad-spectrum antibiotics. Appropriate choice of effective antimicrobial agents for treatment is difficult because of the unpredictability and breadth of antimicrobial resistance of these organisms, which often involves resistance to many of the antibiotics chosen empirically for serious Gram-negative infections.

## Consent

Written informed consent was obtained from the parents of the patient for publication of this case report and any accompanying images. A copy of the written consent is available for review by the Editor-in-Chief of this journal.

## Competing interests

The authors declare that they have no competing interests.

## Authors' contributions

GC and EG were the physicians in charge of our patient throughout his hospitalization and made substantial contributions to conception, acquisition, analysis and interpretation of data and drafting the manuscript. PR and EGG helped to draft the manuscript. MR and AL were involved in revising the manuscript and final approval of the version. All authors read and approved the final manuscript.
